# A semantic segmentation-based automatic pterygium assessment and grading system

**DOI:** 10.3389/fmed.2025.1507226

**Published:** 2025-03-13

**Authors:** Qingbo Ji, Wanyang Liu, Qingfeng Ma, Lijun Qu, Lin Zhang, Hui He

**Affiliations:** ^1^College of Information and Communication Engineering, Harbin Engineering University, Harbin, China; ^2^Key Laboratory of Advanced Marine Communication and Information Technology, Ministry of Industry and Information Technology, Harbin Engineering University, Harbin, China; ^3^School of Public Health, Harbin Medical University, Harbin, China; ^4^School of Medical Humanity, Harbin Medical University, Harbin, China; ^5^Department of Ophthalmology, The Second Affiliated Hospital of Harbin Medical University, Harbin Medical University, Harbin, China

**Keywords:** pterygium, semantic segmentation, deep learning, curve fitting, AI-based diagnostic

## Abstract

**Introduction:**

Pterygium, a prevalent ocular disorder, requires accurate severity assessment to optimize treatment and alleviate patient suffering. The growing patient population and limited ophthalmologist resources necessitate efficient AI-based diagnostic solutions. This study aims to develop an automated grading system combining deep learning and image processing techniques for precise pterygium evaluation.

**Methods:**

The proposed system integrates two modules: 1) A semantic segmentation module utilizing an improved TransUnet architecture for pixel-level pterygium localization, trained on annotated slit-lamp microscope images from clinical datasets. 2) A severity assessment module employing enhanced curve fitting algorithms to quantify pterygium invasion depth in critical ocular regions. The framework merges deep learning with traditional computational methods for comprehensive analysis.

**Results:**

The semantic segmentation model achieved an average Dice coefficient of 0.9489 (0.9041 specifically for pterygium class) on test datasets. In clinical validation, the system attained 0.9360 grading accuracy and 0.9363 weighted F1 score. Notably, it demonstrated strong agreement with expert evaluations (Kappa coefficient: 0.8908), confirming its diagnostic reliability.

**Discussion:**

The AI-based diagnostic method proposed in this study achieves automatic grading of pterygium by integrating semantic segmentation and curve fitting technology, which is highly consistent with the clinical evaluation of doctors. The quantitative evaluation framework established in this study is expected to meet multiple clinical needs beyond basic diagnosis. The construction of the data set should continue to be optimized in future studies.

## 1 Introduction

Pterygium is an abnormal tissue originating from the conjunctiva and growing toward the corneal region (1), typically presenting as pink and wedge-shaped. The majority of these abnormal tissues grow from the inner canthus toward the corneal region, with a minority growing from the outer canthus in the direction of the cornea or bilaterally toward the corneal direction (2). In its early stages, it can cause eye fatigue and dryness (3). As the condition worsens, more abnormal tissue invades the cornea and even the pupil region. In the worst-case scenario, it can lead to blindness, as these tissues obstruct the passage of light through the pupil. The global prevalence of pterygium is 12%, with the lowest rate observed in Saudi Arabia at 0.04% and the highest in Taiwan, China, at 53% (4).

In the early stages of pterygium development, patients can suppress inflammation through the use of medicinal eye drops or delay the invasion of abnormal tissues into the corneal region by reducing exposure to light (5). Beyond a certain point, surgical intervention becomes necessary. Accurate assessment of the condition allows for a timely excision, minimizing damage caused by the operation and reducing recurrence rates (6). Individuals like farmers, fishermen, and workers who work outdoors for prolonged periods and are exposed to sun radiation constitute a high-risk group for developing pterygium (4). Furthermore, those in this demographic often lack access to medical resources, leading to a lack of accurate diagnosis and timely treatment.

With the rapid development of deep learning technologies in recent years, the integration of ophthalmology and artificial intelligence has deepened significantly (7). Many studies have propelled the use of deep learning models in assisting the diagnosis of eye diseases, significantly enhancing diagnostic and treatment efficiencies (8). In the auxiliary diagnosis of pterygium, deep neural networks are primarily used to achieve the following three objectives: (1) classification of pterygium in images, which includes distinguishing between normal eyes and those with pterygium (9–14), as well as categorizing different types of pterygium (15–19); (2) using object detection techniques to locate lesion tissues (11, 20); (3) performing semantic segmentation of lesion tissues at the pixel level (21–23). Specifically, Liu et al. (18) and Wan et al. (19) have based their classification tasks on semantic segmentation results (assessing and grading the severity of pterygium), constructing multi-stage auxiliary diagnostic systems.

The main contributions of this article are summarized as follows:

1. Anterior segment images with pterygium captured by slit lamp photography are collected. The corresponding three regions in the anterior segment image, namely, the cornea, the pupil, and the pterygium, are marked to construct a semantic segmentation dataset of three categories. This provides a data foundation for the auxiliary diagnosis of pterygium.

2. A pterygium assessment and grading system is proposed, utilizing a semantic segmentation network based on convolutional and Vision Transformer architectures to segment three target regions in anterior segment images. The system extracts the contours of the corneal and pupil regions and estimates the depth and region of pterygium invasion into critical regions using an improved curve fitting algorithm. The results are then graded according to clinical standards.

## 2 Materials and methods

**Figure 1:**
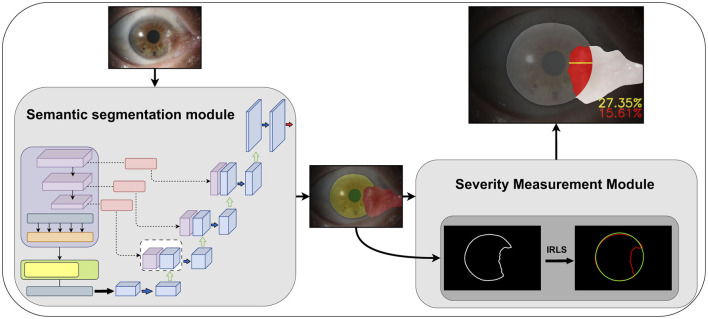
The improved Transunet incorporates a channel and shape attention module (CSAM) into the skip connections.

Figure 1 shows the workflow of our proposed automated pterygium grading system based on anterior segment images. The semantic segmentation module extracts the corneal, pupil, and pterygium regions from the images. The severity assessment module processes these segmented regions using traditional image processing techniques to: (1) quantify the invasion region and depth (expressed as a ratio) of the pterygium within the corneal region; (2) determine whether the pterygium covers the pupil region. The Iteratively Reweighted Least Squares (IRLS) method is primarily employed to robustly fit and reconstruct corneal and pupil regions, demonstrating enhanced robustness against outliers. Final grading integrates these results with clinical expertise.

### 2.1 Dataset

The dataset images in this article are from the Ophthalmology Department of the Second Affiliated Hospital of Harbin Medical University, consisting of 434 anterior segment images containing pterygium of varying degrees. All images have a uniform aspect ratio of 3:2 and a resolution of 2,256 × 1,504 pixels. Anterior segment images are medical images taken with a slit-lamp microscope under diffused light mode and are included in electronic medical records for ophthalmologic diseases. With patient consent, these data were used for this study, with any patient information removed to prevent a breach of privacy.

### 2.2 Segmentation module–Transunet with CSAM

**Figure 2:**
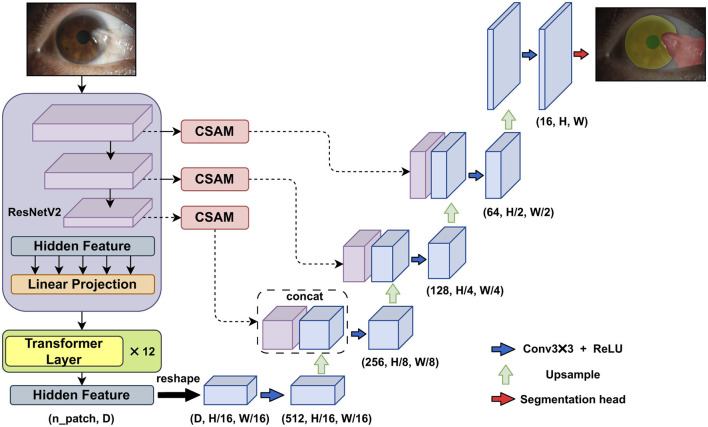
The improved Transunet incorporates a channel and shape attention module (CSAM) into the skip connections.

In the first module of the algorithm's workflow, we improved the Transunet architecture by incorporating the channel and shape attention module (CSAM) into the skip connections, as shown in Figure 2.

The original Transunet architecture includes several main components: ResNetV2 (the first stage of the encoder), Transformer layers (the second stage of the encoder), a convolution-based decoder, and skip connections between the different parts of the network.

In the first stage of the encoder, ResNetV2 is used to downsample the input, generating multi-stage feature maps. The second part of the encoder consists of multiple connected Transformer encoders. The core of each Transformer encoder is the multi-head self-attention mechanism, enabling the Vision Transformer (ViT) to effectively capture long-range semantic relationships within the image.

In the decoder stage, the low-level feature maps are restored to the original input size through consecutive upsampling and skip connections.

#### 2.2.1 Channel and shape attention module (CSAM)

Numerous studies have demonstrated the effectiveness of attention mechanisms in enhancing neural network performance. In the field of computer vision, attention mechanisms improve model performance by guiding the network to focus on the more important features within an image. SENet (24) and CBAM (25) are two representative studies in this region.

**Figure 3:**
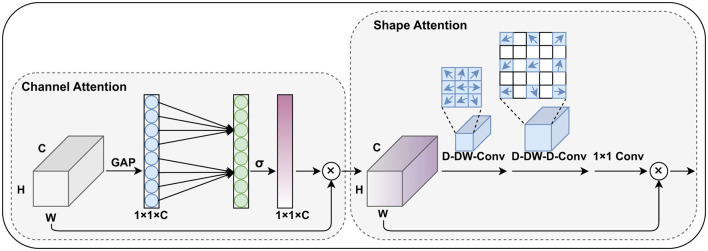
The proposed channel and shape attention module (CSAM), inherits the design principles of CBAM and includes both channel attention and spatial (shape) attention mechanisms.

Based on the concept of generating weights for different channels and spatial positions, this paper improves the CBAM module and proposes the channel and shape attention module (CSAM), as illustrated in the Figure 3.

Similar to CBAM, the CSAM is also divided into two parts: the channel attention module and the spatial attention module. In the channel weight generation stage, we adopted the lightweight design from Wang et al. (26), replacing the fully connected layers with 1D convolutions to capture the relationships between each channel and its neighboring channels. This approach reduces the number of parameters while preserving global information.

In our task, pterygium is a tissue with a relatively clear shape prior, and the feature maps generated by the shallow layers of the encoder contain strong structural information about the target's shape. Research such as Guo et al. (27) and Dai et al. (28) has shown that large convolutional kernels can effectively expand the network's receptive field, and deformable convolutions can effectively capture shape information of the target. Therefore, in the spatial attention module, we combine large convolutional kernels and deformable convolutions to generate spatial position weights for the feature maps, specifically including a deformable depth wise convolution, a deformable depthwise dilated convolution, and a 1 × 1 convolution. This part can be formulated as:


(1) $$\begin{array}{l} \text{Attention}=\text{Conv}1\times 1\left(\text{D-DW-D-Conv}\left(\text{D-DW-Conv}\left({\text{F}}^{\text{′}}\right)\right)\right) \end{array}$$

(2) $$\begin{array}{l} \text{Output}=\text{Attention}⊗{\text{F}}^{\prime } \end{array}$$

F′ represents the feature maps from the skip connections at each stage.

### 2.3 Severity measurement module

**Figure 4:**
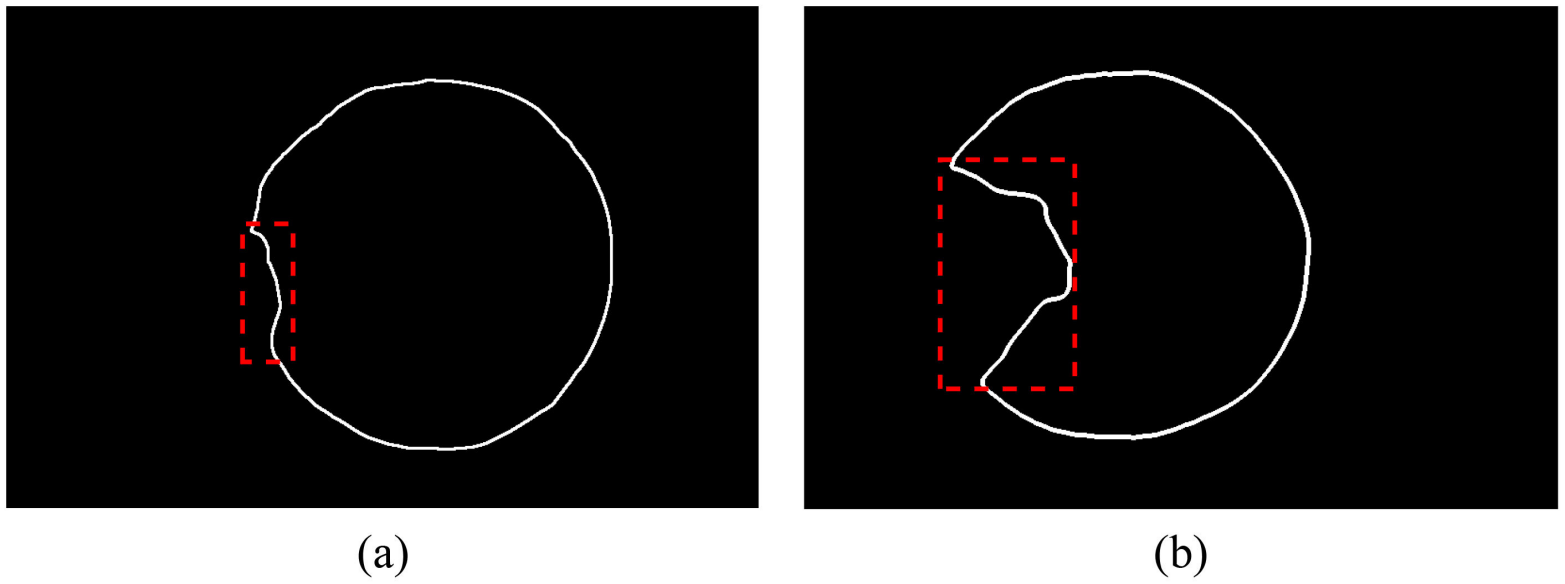
The corneal contour extracted from the segmentation result, and the outliers are shown in the dotted box. **(A)** Pterygium slightly invades the cornea, the proportion of outliers is small. **(B)** Pterygium severely invades the cornea, the proportion of outliers is large.

The contour of the corneal region (or pupil region) is extracted based on the semantic segmentation results, followed by fitting and subsequent calculations. When using ordinary least squares, different degrees of pterygium invasion will affect the fitting results, as shown in Figure 4.

The Iteratively Reweighted Least Squares (IRLS) introduces a distance-weighting function to calculate the weight for each point. After several iterations, the weight of outliers gradually decreases during the fitting process. The weights can be represented as a diagonal matrix, where the values on the diagonal correspond to the weight of each sample.


(3) $$\begin{array}{l} W=\left[\begin{array}{cccc} w_{11} & ... & 0 & 0\\ 0 & w_{22} & ... & 0\\ ... & ... & ... & ...\\ 0 & 0 & ... & w_{mm} \end{array}\right] \end{array}$$

The Huber function and Tukey function are both widely used weighting functions, as shown in Equations 4, 5 respectively. Here, δ represents the residual of a data point, and γ is a hyperparameter.


(4) $$w(\delta )\{\begin{array}{l} 1,\;|\delta |\leq \gamma \\ \frac{\gamma }{\left|\delta \right|},\;|\delta |>\gamma \end{array}$$

(5) $$w(\delta )\{\begin{array}{l} {\left(1-{\left(|\delta |/\gamma \right)}^{2}\right)}^{2},|\delta |\leq \gamma \\ 0\;,\;|\delta |>\gamma \end{array}$$

The formula for calculating the invaded region is as follows:


(6) $$\begin{array}{l} \alpha =\frac{A_{p}}{A_{c}} \end{array}$$

Where *A*_*p*_ represents the region of pterygium covering the corneal or pupil region, and *A*_*c*_ represents the region of the corneal or pupil region (in the same image). *A*_*p*_ and *A*_*c*_ are obtained by performing per-pixel counting of the semantic segmentation results and curve fitting results.

The formula for calculating the invasion depth is as follows:


(7) $$\begin{array}{l} \beta =\frac{\frac{L}{2}-d_{min}}{L} \end{array}$$

**Figure 5:**
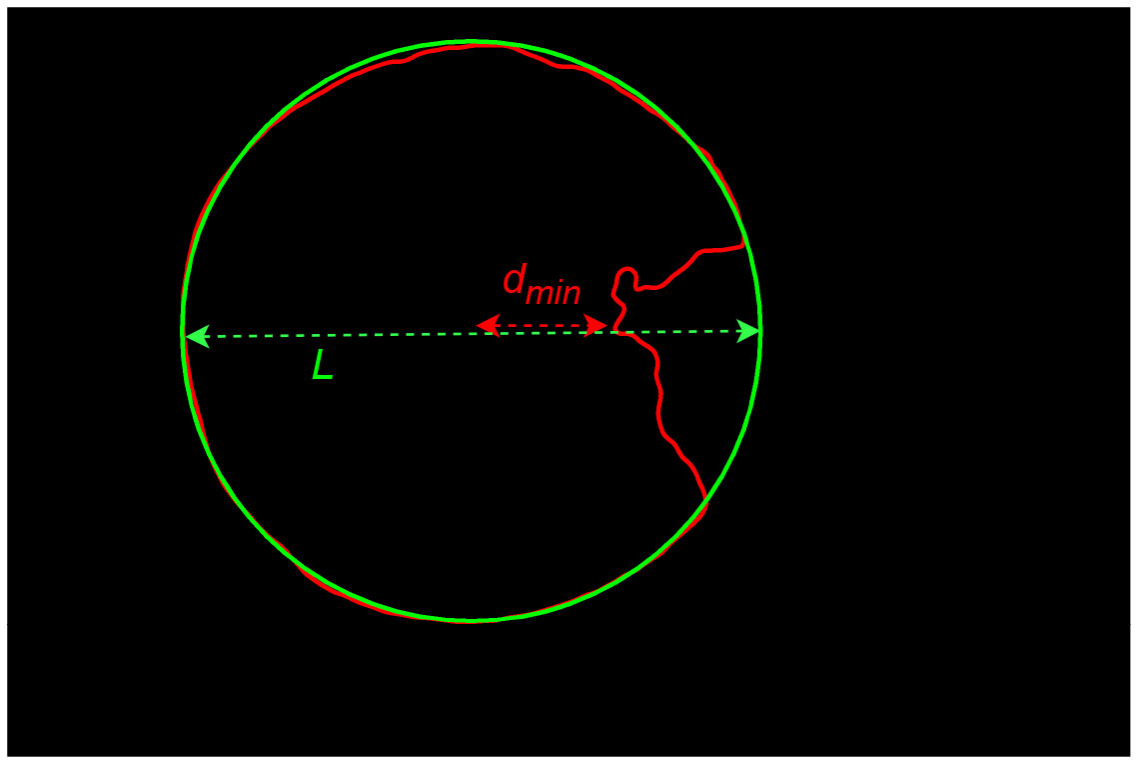
Illustration of the calculation of invasion depth.

Where *d*_*m*_*in* represents the minimum distance from the center of the corneal region (obtained based on the fitting results) to a point on the incomplete corneal contour, and is the corneal diameter in the direction between these two points. As illustrated in Figure 5.

### 2.4 Experiment

**Figure 6:**
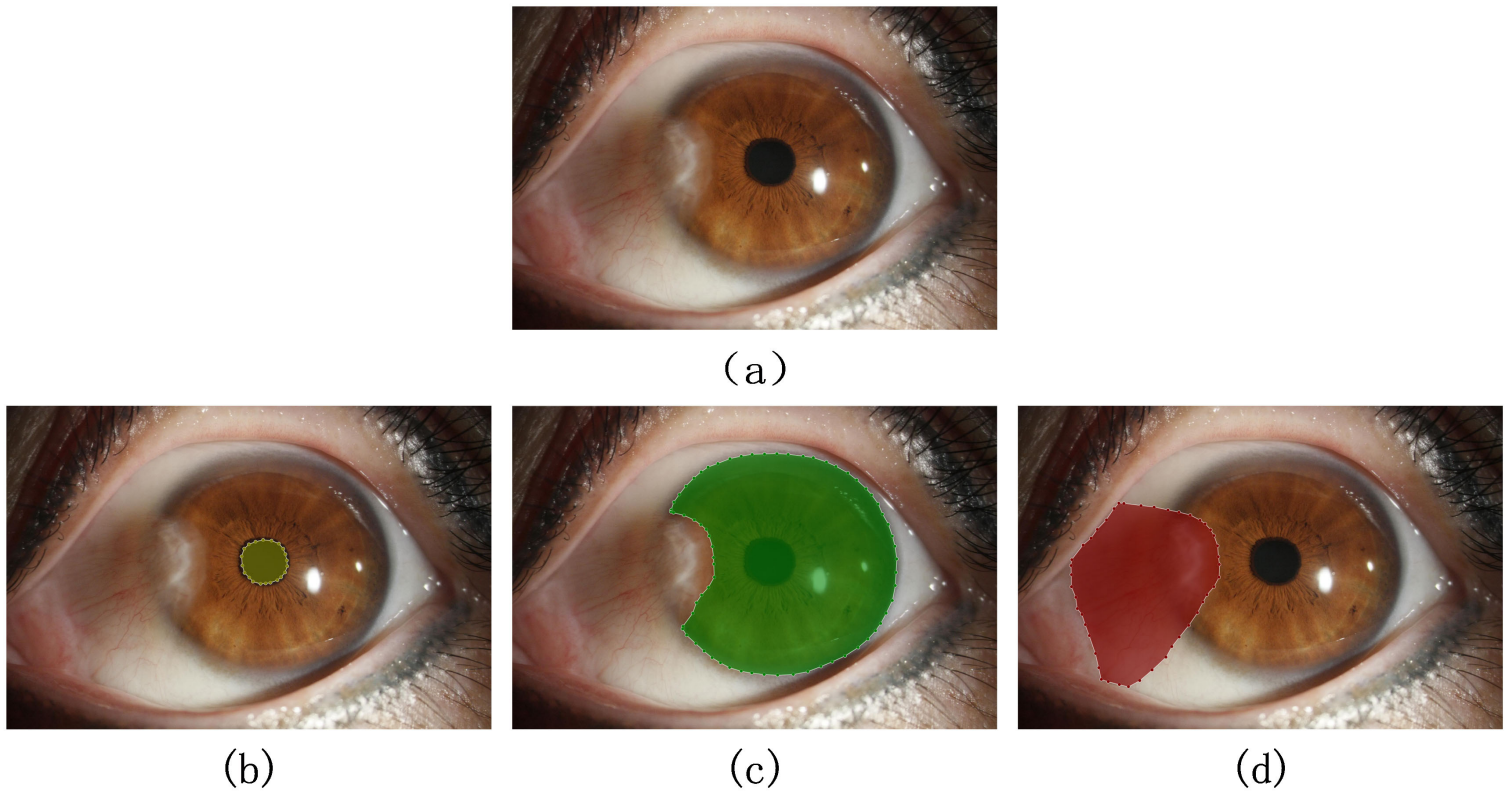
Qualified image and its annotation example: **(A)** original image. **(B–D)** Show the correct marking of the pupil region, corneal region, and the pterygium region, respectively.

To train the semantic segmentation algorithm for automatically segmenting the target categories in images, the original images need to be manually annotated, accurately marking the external contours of the pupil region, corneal region, and pterygium tissue, forming a three-category semantic segmentation dataset, as shown in Figure 6. This process was completed under the guidance of professional physicians.

The dataset was randomly divided into training and testing sets at a ratio of 85:15. The training set consists of 368 images, while the testing set contains 66 images.

We performed data augmentation on the dataset, including random vertical and horizontal flips, as well as color space transformations. During the training phase, we resized all images to 512 × 512 pixels. The backbone of the semantic segmentation network was initialized using the pre-trained weights provided by the authors. Training was conducted on an NVIDIA RTX2080 (Windows system), using the PyTorch framework and the Adam optimizer. The initial learning rate was set to 0.0001, and the training lasted for 150 epochs.

The loss function used for training in this article consists of the cross-entropy loss function and the dice loss function, as illustrated in the following formula:


(8) $$\begin{array}{l} \text{Loss}=\frac{1}{2}\times \text{Cross-Entropy Loss}+\frac{1}{2}\times \text{DiceLoss} \end{array}$$

## 3 Experimental results analysis

### 3.1 Evaluation of the image segmentation module

**Figure 7:**
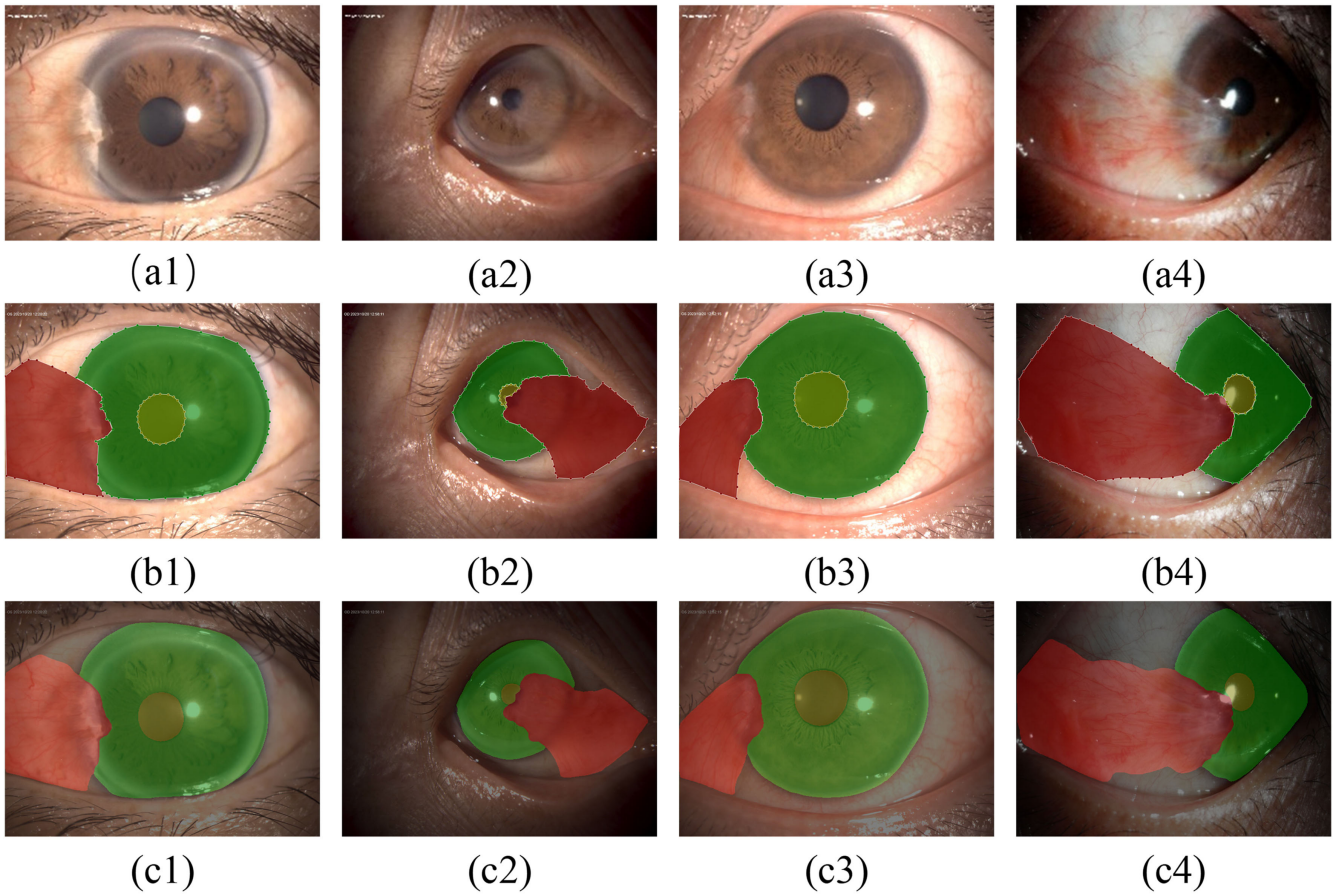
Qualitative evaluation of the segmentation model: **(A1–A4)** show the original images; **(B1–B4)** show the results of annotations under the guidance of experts; **(C1–C4)** show the segmentation results of the model.

In this study, semantic segmentation was performed on the three types of targets present in anterior segment images. Some results are presented in Figure 7.

The test set contains 66 images, and we used a comprehensive set of metrics to evaluate the performance of the segmentation module on the test set. These metrics include Intersection over Union (IoU), Dice Coefficient (DSC), Precision, and Recall, which are calculated as follows (for individual categories):


(9) $$\begin{array}{l} \text{Precision}=\frac{TP}{TP+FP} \end{array}$$

(10) $$\begin{array}{l} \text{Recall}=\frac{TP}{TP+FN} \end{array}$$

(11) $$\begin{array}{l} \text{IOU}=\frac{TP}{TP+FP+FN} \end{array}$$

(12) $$\begin{array}{l} \text{Dice}=\frac{2\times TP}{2\times TP+FP+FN} \end{array}$$

where TP refers to the true positive part of the category, FP is the false positive part, and FN is the false negative part.

To evaluate the performance of the model on the test set, the aforementioned metrics are used to perform a quantitative analysis of the segmentation module's results, which are shown in Table 1.

**Table 1:** Performance of the Transunet on the test set(%).

**Category**	**IOU**	**Recall**	**Precision**	**Dice-score**
Pterygium	82.50	91.11	89.73	90.41
Pupil region	94.09	96.11	97.81	96.95
Cornea region	95.33	97.75	97.47	97.61

Here CSAM is used as an attention module to enhance the features in the skip connections. Therefore, we compared its performance with other classic attention modules commonly used in the medical image segmentation field, as shown in Table 2 (below each category are the IOU metrics for that category).

**Table 2:** Performance of different models on the test set(%).

**Method**	**mIOU**	**Avg dice**	**Pterygium**	**Pupil region**	**Cornea region**
Transunet (29)	90.22	94.74	81.24	**94.25**	95.19
Transunet+attention gate (30)	89.14	93.89	80.14	93.94	93.35
Transunet+SENet	90.01	94.80	81.06	93.54	**95.45**
Transunet+CBAM	89.69	94.47	82.00	92.05	95.05
Transunet+CSAM (ours)	**90.59**	**94.89**	**82.50**	94.09	95.19

Bold values indicate the best results among different methods.

Compared to other attention modules, the attention module we proposed achieves better improvements.

According to Tables 1, 2 and Figure 7, the model has achieved high accuracy in segmenting the pupil and corneal regions, while the segmentation performance for pterygium is influenced by its specific morphology. Pterygium that is raised with well-defined edges yields better segmentation results, whereas the performance is poorer for less distinct edges, as shown in Figure 7C4.

For the portion of the pterygium invading the corneal limbus, where the edges are clearer, the segmentation results are more accurate. This clarity is beneficial for the post-processing of the segmentation results.

### 3.2 Severity assessment module analysis

**Figure 8:**
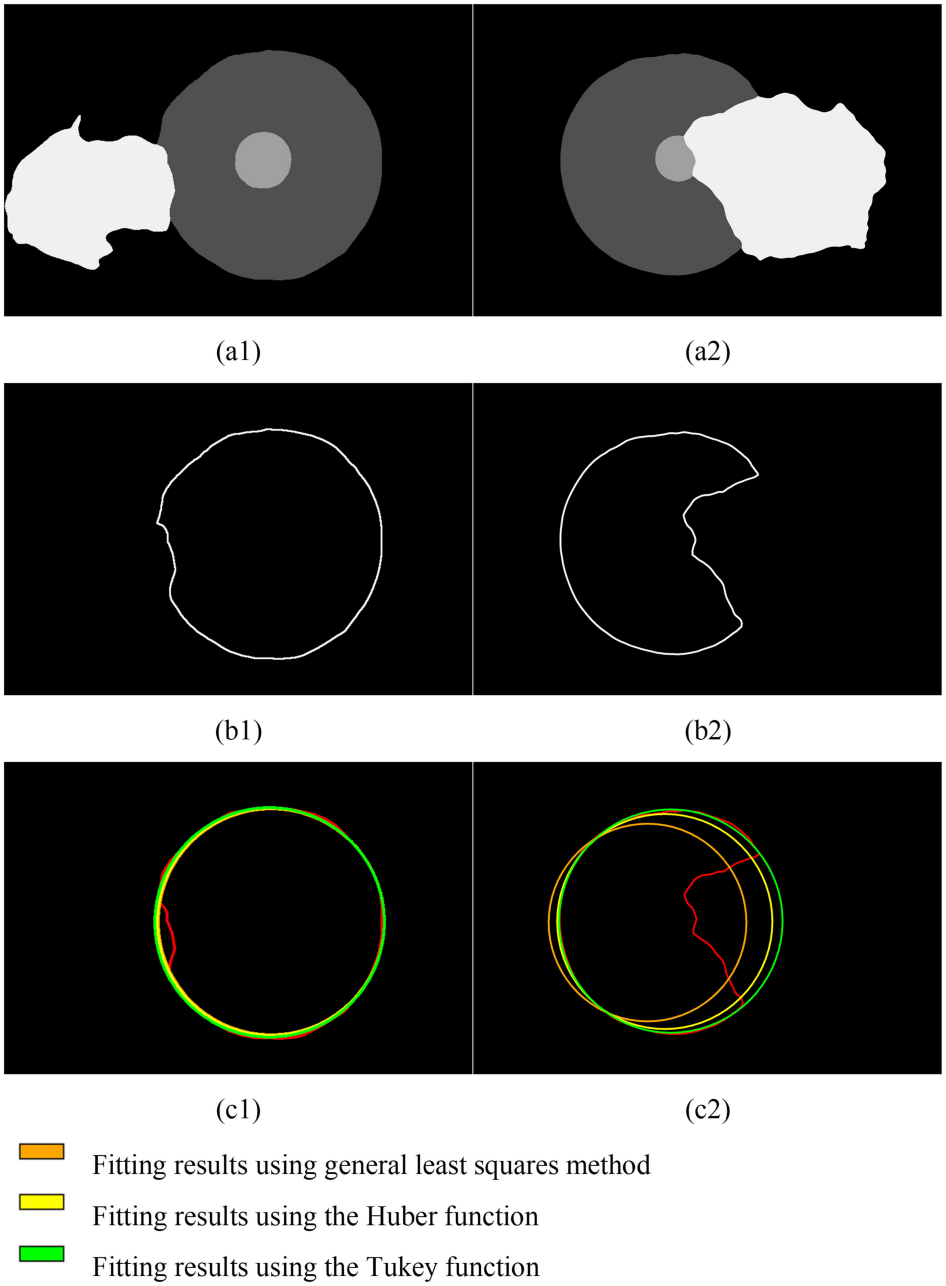
Fitting results of the corneal contour based on semantic segmentation for pterygium of different severity levels. **(A1, A2)** Results of semantic segmentation; **(B1, B2)** corneal contours extracted based on semantic segmentation results; **(C1, C2)** fitting results.

In this study, we used the Iterative Reweighted Least Squares (IRLS) method to fit and restore the incomplete corneal region extracted via semantic segmentation. We tested two weight functions—the Huber function and the Tukey function—and conducted a total of ten iterative processes, comparing the results with those from the general least squares method. The results are shown in the Figure 8.

Due to the varying depth of pterygium invasion into the corneal limbus, the Iteratively Reweighted Least Squares (IRLS) method using the Tukey weight function achieved the best fitting results under comprehensive conditions.

**Figure 9:**
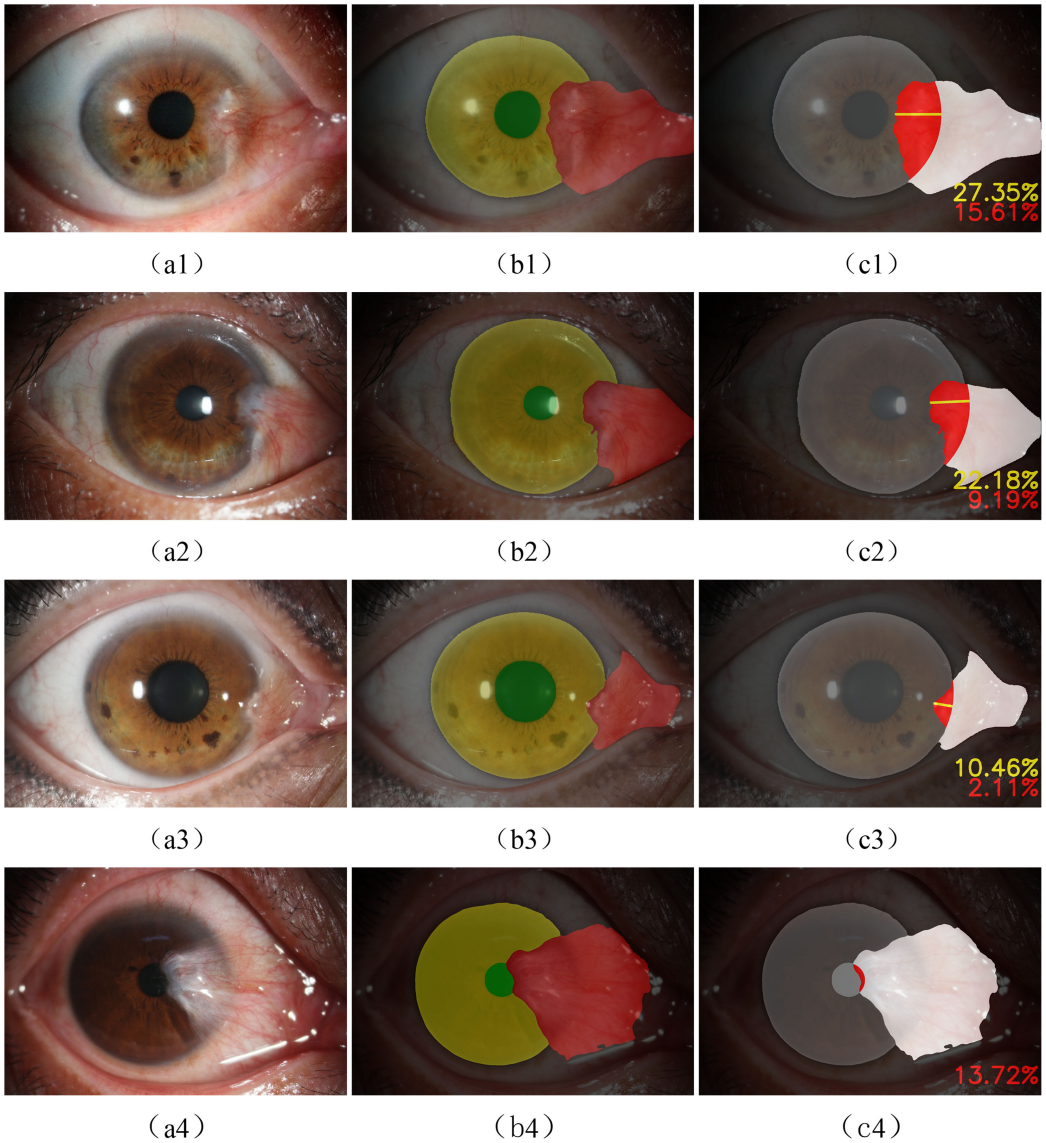
**(A1–A4)** the original anterior segment image, **(B1–B4)** the result of semantic segmentation, and **(C1–C4)** the outputs of the evaluation module.

The output of the evaluation module based on the IRLS using the Tukey weight function is shown in Figure 9. Figures 9A1–A3 depict typical cases where the pterygium covers the corneal surface but does not obscure the pupil, with results showing the depth and region (in percentage) of its invasion into the corneal region. Figure 9A4 presents a more severe case of pterygium, where the pterygium has already covered the pupil (with the invasion region percentage also calculated). All of these results are derived from images in the test set.

### 3.3 Overall reliability analysis of the system

Clinically, pterygium can be classified into three grades. Grade 1: pterygium invades the cornea ≤ 3 mm; Grade 2: pterygium invades the cornea >3 mm but does not reach the pupil margin; Grade 3: pterygium has covered the pupil (as shown in Figure 9A4).

The algorithm is presented externally as a classification model. To evaluate the reliability of the proposed method for pterygium grading, we additionally collected anterior segment images containing pterygium from 125 cases. These images were graded by experienced ophthalmologists, and the number of pterygium cases with varying severity levels is shown in Table 3.

**Table 3:** Composition of the dataset used for method evaluation.

**Type**	**Grade 1**	**Grade 2**	**Grade 3**
Number	46	65	14

**Figure 10:**
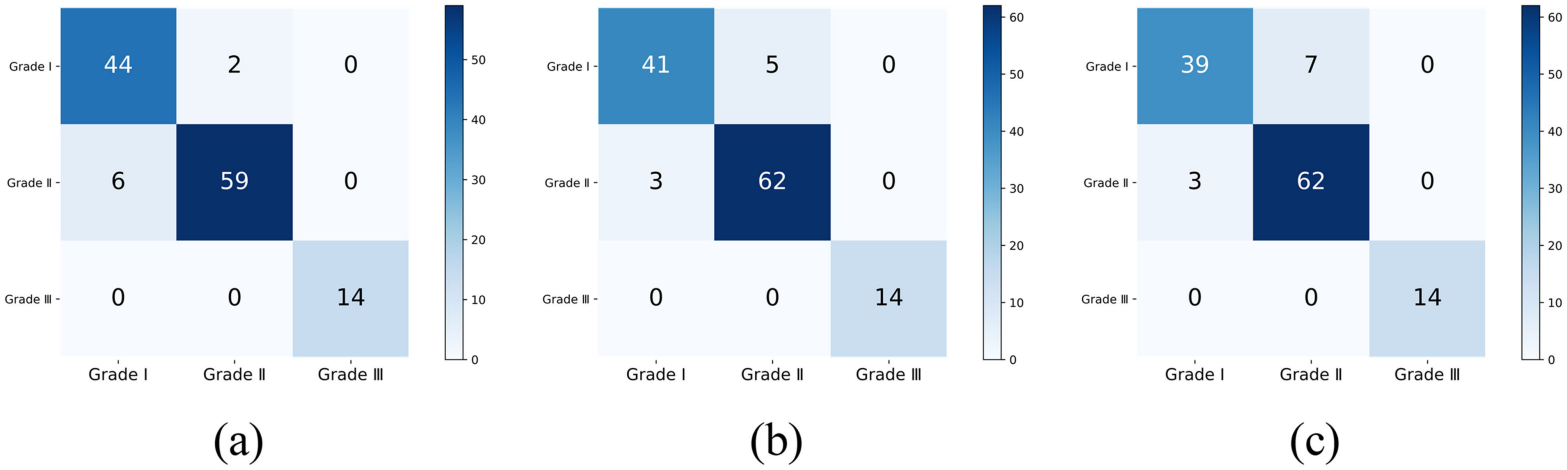
The classification confusion matrices under different reference diameters: **(A)** L = 11.5 mm, **(B)** L = 12.0 mm, **(C)** L = 12.5 mm.

The common range of adult corneal diameters is 11.5 mm to 12.5 mm. Within this range, we selected a reference diameter at intervals of 0.1 mm. The confusion matrices of the classification results, when the reference diameters L are 11.5 mm, 12.0 mm, and 12.5 mm, are shown in Figure 10 (different reference diameters mainly affect the distinction between Grade 1 and Grade 2).

We use accuracy to measure the overall classification performance, and the weighted F1 score and Kappa consistency coefficient to evaluate the impact of class imbalance. Their calculation expressions are as follows:


(13) $$\begin{array}{l} \text{Accuracy}=\frac{TP+TN}{TP+TN+FP+FN} \end{array}$$

(14) $$\begin{array}{l} \text{Weighted F1 Score}=\overset{n}{\underset{i=1}{\sum }}w_{i}\times F1_{i},F1_{i}=2\times \frac{\text{Precision }\times \text{ Recall}}{\text{Precision }+\text{ Recall}} \end{array}$$

*F*1_*i*_ is the F1 score of the *i*-th class, and *w*_*i*_ is the proportion of samples in that class.


(15) $$\begin{array}{l} \kappa =\frac{P_{o}-P_{e}}{1-P_{e}} \end{array}$$

Where *P*_*o*_ is the actual classification accuracy, and *P*_*e*_ is the expected accuracy.

The classification results for all reference diameters are shown in the Table 4.

**Table 4:** The classification metrics under different reference diameters.

**Reference diameter (mm)**	**Accuracy**	**Weighted F1 Score**	**Kappa consistency coefficient**
11.5	0.9360	0.9363	0.8908
11.6	0.9360	0.9363	0.8908
11.7	0.9360	0.9357	0.8895
11.8	0.9360	0.9357	0.8895
11.9	0.9360	0.9357	0.8895
12.0	0.9360	0.9357	0.8895
12.1	0.9360	0.9357	0.8895
12.2	0.9280	0.9275	0.8754
12.3	0.9280	0.9275	0.8754
12.4	0.9280	0.9275	0.8754
12.5	0.9200	0.9193	0.8754

For the dataset used in this study, 11.5 mm may be the most optimal reference diameter, with an accuracy of 0.9360, a weighted F1 score of 0.9363, and a Kappa consistency coefficient of 0.8909. This indicates that the grading results of the proposed method have a high level of agreement with the physicians' grading results.

The method proposed in this paper achieves quantitative calculation of the extent of pterygium invasion into critical regions. Based on semantic segmentation and curve fitting, it performs well in tasks such as determining the size and location of the pterygium and assessing its severity. In primary healthcare units and rural regions, clinicians can use it as an auxiliary tool to quantify pterygium progression and standardize severity evaluation. By integrating personal experience and patient history, it enhances the diagnostic accuracy of pterygium.

## 4 Discussion

In this study, we aimo utilize a deep learning model trained on anterior segment images, supplemented by traditional image analysis methods, to achieve automated measurement of pterygium progression.

The key to training the deep learning model lies in acquiring a high-quality dataset. The slit lamp, an effective tool for ophthalmic examinations, can capture high-resolution images with rich details. However, this does not entirely eliminate the limitations in dataset construction. In this study, all anterior segment images were obtained from the same device model, and the majority of subjects were from the same region. Such a dataset may limit the model's generalizability. Future research should focus on enriching the dataset, such as collecting images from patients in diverse regions and incorporating images captured by smartphones and cameras to enhance the model's generalization performance. In studies (21–23), the Intersection over Union (IoU) metrics for pterygium segmentation were 83.8%, 86.4%, and 78.1%, respectively. However, due to differences in datasets, it is challenging to objectively compare the performance of different segmentation models. Additionally, this method has other limitations. Compared to Hilmi et al. (31), who considered the degree of redness in pterygium, this study only utilized information related to the size and location of the target in anterior segment images, without incorporating other valuable features such as color and transparency of the pterygium. The lack of utilization of patient medical history also reduces the reliability of auxiliary diagnosis.

This study references medical prior knowledge, assuming a corneal diameter of 12 mm to calculate the actual invasion depth and approximating the corneal region as a circular shape without considering curvature or other shape parameters. While this simplification facilitates computation, it introduces additional errors. Future work should seek more accurate region calculation methods and account for individual differences among patients to optimize diagnostic logic.

## 5 Conclusion

Pterygium is a common ocular surface disorder characterized by abnormal tissue growth from the conjunctiva toward the cornea. Surgical removal is required when the pterygium encroaches significantly onto the corneal region. Accurate assessment of its pathological progression is essential for determining treatment strategies. This paper proposes an automated pterygium evaluation method that integrates deep learning models with traditional image processing algorithms, aiming to optimize diagnostic workflows and enhance efficiency. We utilized patient images captured by slit-lamp microscopy from the Ophthalmology Department of the Second Affiliated Hospital of Harbin Medical University, annotated as a dataset for model training and testing. The system was further evaluated on additional patient images to validate its effectiveness.

This study establishes a standardized quantitative assessment framework for pterygium, which is expected to address multiple clinical needs beyond basic diagnosis: (1) Longitudinal monitoring of disease progression. This is particularly crucial in telemedicine scenarios for determining the timing of surgical intervention and monitoring treatment efficacy; (2) Automated measurement significantly reduces inter-observer variability inherent in manual assessments, ensuring consistency in evaluation; and (3) By enabling digital recording and standardized grading criteria, the framework supports large-scale screening programs, facilitates extensive epidemiological studies, and allows for cross-population analysis of disease progression characteristics.

Although slit-lamp examination remains the gold standard for diagnosis, our automated system specifically addresses challenges in quantitative analysis and assessment standardization, proving particularly valuable in telemedicine settings and regions with limited specialist resources.

## Data Availability

The data analyzed in this study is subject to the following licenses/restrictions: the dataset images are exclusively sourced from the Second Affiliated Hospital of Harbin Medical University, which may limit the generalizability of the findings to other regions or institutions. The dataset contains anterior segment images with varying degrees of pterygium, which might limit the statistical power and robustness of models trained on this dataset, particularly for rare or extreme cases. All images were captured using a slit-lamp microscope in diffuse illumination mode. This specific imaging condition may restrict the applicability of the dataset to other imaging techniques or settings. The segmentation labels for pupil, cornea, and pterygium regions were manually annotated under the guidance of professional ophthalmologists. Although expert-guided, manual annotation can introduce subjectivity and potential inter-observer variability, affecting the consistency of the dataset. While patient information was removed to protect privacy, the dataset is limited to cases where patient consent was obtained. This might exclude certain patient demographics and could introduce selection bias. Requests to access these datasets should be directed to Lijun Qu, qulijun@hrbmu.edu.cn.
